# NAD^+^ Homeostasis and NAD^+^-Consuming Enzymes: Implications for Vascular Health

**DOI:** 10.3390/antiox12020376

**Published:** 2023-02-04

**Authors:** Roberto Campagna, Arianna Vignini

**Affiliations:** 1 Department of Clinical Sciences, Polytechnic University of Marche, 60100 Ancona, Italy; 2 Research Center of Health Education and Health Promotion, Università Politecnica delle Marche, 60121 Ancona, Italy

**Keywords:** nicotinamide adenine dinucleotide, NAD^+^, endothelium, vascular health, SIRT1, PARP1, NNMT

## Abstract

Nicotinamide adenine dinucleotide (NAD^+^) is a ubiquitous metabolite that takes part in many key redox reactions. NAD^+^ biosynthesis and NAD^+^-consuming enzymes have been attracting markedly increasing interest since they have been demonstrated to be involved in several crucial biological pathways, impacting genes transcription, cellular signaling, and cell cycle regulation. As a consequence, many pathological conditions are associated with an impairment of intracellular NAD^+^ levels, directly or indirectly, which include cardiovascular diseases, obesity, neurodegenerative diseases, cancer, and aging. In this review, we describe the general pathways involved in the NAD^+^ biosynthesis starting from the different precursors, analyzing the actual state-of-art of the administration of NAD^+^ precursors or blocking NAD^+^-dependent enzymes as strategies to increase the intracellular NAD^+^ levels or to counteract the decline in NAD^+^ levels associated with ageing. Subsequently, we focus on the disease-related and age-related alterations of NAD^+^ homeostasis and NAD^+^-dependent enzymes in endothelium and the consequent vascular dysfunction, which significantly contributes to a wide group of pathological disorders.

## 1. Introduction

Nicotinamide adenine dinucleotide (NAD^+^) plays a crucial role in many vital cellular reactions and functions, including cell energy production, cellular metabolism, and survival. NAD^+^ takes part in redox reactions, where the reduced form of NAD^+^, NADH, acts as an electron donor in redox reactions, and serves as co-substrate in many other reactions [1,2]. The NAD^+^ concentration, in healthy mammalian cells, is nearly 0.0004–0.0005 mol/L, and its half-life is approximately 1 h [3].

Since NAD^+^ levels can directly and indirectly impact a number of metabolic functions regulated by enzymes whose activity depends on NAD^+^, changes in intracellular NAD^+^ levels affect all the processes regulated by these enzymes, which include cellular metabolism, gene expression regulation, DNA repair, mitochondrial functions, redox reactions, inflammation, intracellular trafficking, aging, and cell death [4,5]. Therefore, many pathological conditions are associated with an impairment of intracellular NAD^+^ levels, including cardiovascular diseases, cancer, obesity, neurodegenerative diseases, and aging [6,7,8].

In the context of vascular diseases, the administration of NAD^+^ precursors or the inhibition of NAD^+^-consuming enzymes may ensure proper vascular health, thus improving conditions characterized by cardiovascular and cerebrovascular disorders in the elderly. This is particularly of interest, as these vascular diseases frequently co-exist. In fact, age-related alterations in endothelium and the consequent vascular dysfunction significantly contribute to a wide group of pathological disorders associated with old age [9,10].

Vascular diseases are the pathologies with the most important epidemiological impact due to longer lifespans and, consequently, to the general increase in the average age. Beyond its role as a physical barrier, the endothelium is nowadays recognized as a dynamic and multi-functional endocrine organ, playing critical roles in maintaining homeostasis, including blood filtration, vessel tone adjustment, hemostasis, immune response modulation, hormone trafficking, and angiogenesis. Therefore, keeping the vascular vessels healthy is fundamental for the homeostasis of the whole body [11]. The endothelial dysfunction represents the first step in vascular alteration, which leads to a vasoconstrictive, pro-thrombotic, and pro-inflammatory state, as well as changes in the extracellular matrix. All these changes greatly impact on vascular stability. Indeed, vascular alterations represent the first step in developing cardio- and cerebro-vascular diseases, including atherosclerosis, stroke, or coronary artery disease, which are all well-known causes of disability and mortality for the older population [12].

This review focuses on NAD^+^ homeostasis, metabolism, and the impact of NAD^+^ levels and NAD^+^-depending enzymes on vascular function.

## 2. NAD^+^ Biosynthesis

**Figure 1:**
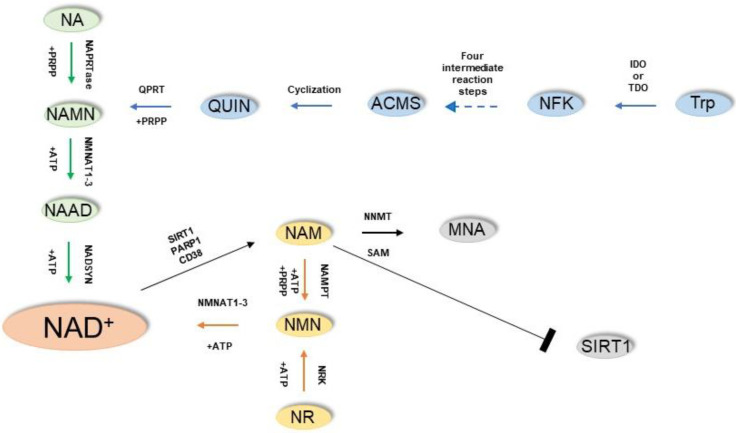
Pathways of NAD^+^ biosynthesis and major NAD^+^-consuming enzymes. The Preiss–Handler pathway is highlighted in green; the de novo biosynthesis pathway is in blue; the NAD^+^ salvage pathway is in yellow.

The NAD^+^ can be synthesized starting from four main molecules, from which different metabolic pathways drive to the yield of the same product, underlying its vital role in the cell (Figure 1). These molecules are the amino acid tryptophan (Trp), nicotinic acid (NA), nicotinamide (NAM), and nicotinamide riboside (NR) [13].

The Preiss–Handler pathway is responsible for the formation of NAD^+^ starting from NA. In this pathway, the enzyme nicotinic acid phosphoribosyltransferase (NAPRTase) catalyzes the conversion of NA and 5-phosphoribosyl-1-pyrophosphate (PRPP) to nicotinic acid mononucleotide (NAMN) and pyrophosphate (PPi). Subsequently, the enzymes nicotinamide mononucleotide adenylyltransferases1-3 (NMNATs1-3) convert the NAMN into NA adenine dinucleotide (NAAD), which in turn is finally converted into NAD^+^ by the enzyme NAD synthase (NADSYN) following an amidation reaction.

The synthesis of NAD^+^ can also start from the Trp, converting the aminoacid into N-formylkynurenine either by indoleamine 2,3-dioxygenase (IDO) or tryptophan 2,3-dioxygenase (TDO). Following four intermediate reaction steps, N-formylkynurenine (NFK) is ultimately converted into α-amino-β-carboxymuconate-ε-semialdehyde (ACMS), an unstable molecule which cyclizes spontaneously to quinolinic acid (QUIN). Finally, the enzyme quinolinate phosphoribosyltransferase (QPRT) utilizes the 5-phosphoribosyl-1-pyrophosphate as a co-substrate to catalyze the conversion of quinolinic acid into NAMN, which is finally converted into NAD^+^ through the remaining reactions of the Preiss–Handler pathway. The biosynthesis of NAD^+^ starting from NAM is known as the “NAD^+^ salvage pathway” and is based on the conversion of NAM to NMN by the enzyme nicotinamide phosphoribosyltransferase (NAMPT) using ATP and PRPP as co-substrate, and subsequently the NMNAT enzymes catalyze the conversion of NMN to NAD^+^. Finally, NAD^+^ can be produced starting by NR, which is phosphorylated by the NR kinases (NRK1/NRK2) yielding NMN, which is converted to NAD^+^ by NMNAT enzymes.

The term niacin or vitamin B3 comprises the NA and NAM, both precursors of NAD^+^, and the ingestion of a quantity equal or slightly less than 20 mg of niacin is considered sufficient for the daily requirements for NAD^+^ biosynthesis [4]. Indeed, the so-called pellagra, a condition caused by low consumption of niacin or tryptophan, became rare in developed countries and mostly is associated with tuberculosis, alcoholism, or eating disorders which interfere with the absorption of the vitamin, while it remains endemic in underdeveloped countries [14].

NMN and NR are present in a broad variety of foods, including fruits, vegetables, and meat, and recently their presence has been demonstrated in milk, although only at micromolar concentrations [15].

Many studies have demonstrated that the administration of NMN is able to boost the NAD^+^ biosynthesis in various peripheral organs and tissues. Furthermore, it has been recently reported that a long-term oral supplementation (one year) of NMN up to 300 mg/kg is safe, well-tolerated, and does not trigger any harmful or toxic impacts in mice [16,17,18]. Bieganowski and Brenner were the first to report a direct involvement of the NR in the NAD^+^ metabolism [19].

The first reaction catalyzed by NAMPT in the NAD^+^ salvage pathway is rate-limiting, energetically expensive, and exposed to feedback inhibition by NAD^+^. Thus, NR is an interesting player which could be able to boost NAD^+^ levels beyond what is attainable via the conventional B vitamin metabolism. [20,21,22]. Among NAD^+^ precursors, Trp seems to be less efficient in boosting NAD^+^ biosynthesis, since it was demonstrated that Trp supplementation should reach a range of 34–86 mg of Trp in order to provide the equivalent of 1 mg of niacin [23].

## 3. NAD^+^ Use: Redox Reactions and NAD^+^-Dependent Enzymes

Glycolysis is the best-known pathway in which NAD^+^ exerts a fundamental role. Indeed, two NAD^+^ molecules per molecule of glucose are needed during the conversion of glucose to pyruvate in the process of glycolysis. Upon converting a molecule of glucose into two molecules of glyceraldehyde-3-phosphate (G3P), the enzyme glyceraldehyde-3-phosphate dehydrogenase (GAPDH) reduces NAD^+^ to NADH to trigger the conversion of a molecule of G3P into a molecule of 1-3-biphosphoglycerate [24].

Another crucial role played by NAD^+^ is in the tricarboxylic acid (TCA) cycle since, upon entering the mitochondrion, the TCA cycle reduces NAD^+^, yielding NADH molecules. The mitochondrial NADH molecules, which have been generated from glycolysis or the TCA cycle, were then oxidized by the Complex I of the electron transport chain [24]. Thus, both glycolysis and the TCA cycle can affect the NAD^+^ homeostasis by modifying the NAD^+^ and NADH contents in the cytoplasm and the nucleus, respectively [25].

Interestingly, it was demonstrated that aging is accompanied by a decrease of NAD^+^/NADH ratio in human plasma, attributable to a depletion of NAD^+^ stores rather than consequences of an increase of NADH content [26]. In recent years, the functions of NAD^+^ have been elucidated beyond its role as a coenzyme since NAD^+^ and its metabolites also represent degradation substrates for a broad range of enzymes [27]. In the next paragraphs, we will focus on the NAD^+^-dependent enzymes mostly involved in vascular aging and function.

## 4. NAD^+^ and Vascular Function

Vascular aging is the result of a gradual change of vascular structure and function in the blood vessels and plays a central role in both cardiovascular and cerebrovascular diseases. In vascular aging, the primary event is an impaired vasodilatation and abnormal vasoconstriction as result of endothelial dysfunction [28,29,30,31].

It has been hypothesized that aging and chronic systemic inflammation could play a key role in the development of many diseases, including cardiovascular disorders [32,33,34,35,36,37,38]. In line with this hypothesis, it has been proposed that the reduction in NAD^+^ content, due to aging, could be linked to a low-grade chronic inflammation. Indeed, in a recent work, it was found that senescent cells are able to stimulate the proliferation of proinflammatory ex vivo mouse macrophages characterized by an overexpression of CD38. CD38 is a multifunctional enzyme that utilizes NAD^+^ as a substrate to generate second messengers and is considered one of the main modulators of cellular NAD^+^ levels in mammals. Consistently, these macrophages accumulate in metabolic tissues, including liver and visceral white adipose tissue, during ageing and acute responses to inflammation, contributing to the age-dependent NAD^+^ decline [39]. CD38 is known to be expressed at very high levels in endothelium, but its upregulation has also been detected in human macrophages during inflammation, as well as in blood samples collected from older adults, and thus it has been proposed that age-related inflammation could be counteracted by boosting NAD^+^ synthesis or fighting NAD^+^ depletion [40,41,42,43]. In order to boost the NAD^+^ synthesis, the possibility of achieve this result by administering NAM was explored, which is the precursor of NAD^+^, although high doses of NAM act as a SIRT1 inhibitor, dramatically reducing lifespan [44,45]. In in vivo high-fat diet aged mice chronic supplementation of NAM was able to induce a marked reduction of the inflammation and ameliorated their healthspan without extending lifespan [46]. Similarly, another study demonstrated that NAM administration significantly inhibited the secretion of key inflammatory cytokines and cyclooxygenase (COX)-derived metabolites following the differentiation of monocytes into low-inflammatory phenotype macrophages, while the administration of niacin, another NAD^+^ precursor, promoted cardiac healing after myocardial infarction by stimulating M2 polarization of peripheral monocytes in vitro [47,48]. Moreover, in hypertensive mice characterized by a genetic or pharmacologically-induced dysfunction of endothelial nitric oxide synthase, NAM supplementation in vivo was demonstrated to decrease renal mRNA levels of inflammatory markers and decrease arterial blood pressure. These findings suggested that NAM supplementation could be useful to hypertensive patients characterized by an impaired endothelial NO synthase (eNOS) system where the suppression of inflammation could be specifically efficient [49,50]. Indeed, inflammation plays a key role in many pathological processes favoring cell proliferation, migration, and differentiation.

Since NMN and NR are also NAD^+^ precursors, the administration of these molecules was also studied aiming anti-inflammatory effects both in in vitro and in vivo models [13,51]. A recent study demonstrated that NMN and NR administration to endothelial cells suppressed the inflammation induced by IL-1β and TNF-α. Furthermore, in the same study it was demonstrated that NMN and NR administration ameliorated the angiotensin II-induced impairment of endothelium-dependent vasodilation in ex vivo murine aortic rings [40]. The administration of NMN was able to revert the endothelial dysfunction and inflammation by extracellular conversion to NR through CD73, an enzyme present on the luminal surface of endothelium, although the vasoprotective effects observed upon NR administration were CD73-independent, an involvement of SIRT1 has been hypothesized [40]. The implications of SIRT1 activity on endothelial ageing and function are important and largely studied, therefore a following paragraph of this review will be dedicated to this topic.

The fatty acid β-oxidation and the oxidative phosphorylation, which occurs in mitochondria, are largely influenced by the intracellular NAD^+^ levels, thus the progressive decline of NAD^+^ levels greatly impact the mitochondrial function in the endothelial cells, as demonstrated by several studies in which the administration of NAD^+^ precursors molecules reduced the oxidative stress of mitochondria and reverted the related vascular dysfunction in vitro and in vivo [1,52,53]. In aged mice, the chronic administration of NMN triggered a decrease in the aortic pulse wave, propagating velocity and partially reverting the NO-related endothelial dysfunction, an effect that was associated with the decrease of the collagen accumulation rate and with an increased deposition of elastin, while a decrease in oxidative stress was also observed [52].

In line with these findings, chronic NMN supplementation normalized the amount of reactive oxygen species (ROS) produced by mitochondria, improving the mitochondrial bioenergetics and rescuing the cerebromicrovascular endothelial function and neurovascular coupling responses in aged mice [54]. Accordingly, in a model of aged mice aorta, the administration of NMN was able to revert the changes observed in the expression of microRNA patterns, which correlated with boosted biogenesis of mitochondria [55].

In another in vivo study, it was reported that the chronic administration of NMN in mice exerted neurovascular protective effects due to the activation of transcription of genes that play a key role in rejuvenation of mitochondria and in anti-inflammatory and anti-apoptotic pathways [56].

Interestingly, in vivo NMN administration to mice, alone and in combination with exogenous hydrogen sulfide, was also proved to enhance skeletal muscle blood flow by decreasing the age-associated diminution in capillary density through the activation of vascular endothelial growth factor signaling in a SIRT1-dependent manner [57].

While the supplementation with NAD^+^ precursors is a promising strategy to restore NAD^+^ levels, several studies explored whether blocking NAD^+^-consuming enzymes could be an effective strategy to revert the NAD^+^ decline [58,59].

In this regard, numerous studies focused on the inhibition of cyclic ADP-ribose synthase CD38, a NAD^+^ consuming enzyme abundant in endothelial cells whose expression is intensely activated by hypoxia-reoxygenation resulting in loss of eNOS-mediated nitric oxide generation and overstressed eNOS uncoupling. The natural molecule apigenin, which belongs to the flavonoid group, was demonstrated to inhibit CD38 activity, leading to an increase in NAD^+^ levels and decreased global acetylation in cell cultures. In an in vivo model of aged mice, oral apigenin administration reverted vascular endothelial dysfunction and large elastic artery stiffening, two clinically important signs of arterial dysfunction with age, and also inhibited the foam cell formation in an established cell culture model of early atherosclerosis [60]. Thus, taken together, these preclinical evidences offer an experimental basis for further translational studies in order to evaluate the effective potential of apigenin to treat arterial dysfunction and reduce cardiovascular disease risk with aging, due to its CD38 inhibition activity.

## 5. NAD^+^-Dependent Enzymes and Vascular Function: SIRT1

Sirtuins are NAD^+^-dependent deacetylases enzymes that catalyze the removal of acetyl group from lysines on histones and proteins, yielding NAM and O-acetyl-ADP-ribose [61]. However, while SIRT1, SIRT2, and SIRT3 display an intense deacetylase activity, SIRT4, SIRT5, and SIRT6 exhibit a weaker deacetylase activity. Sirtuins play a key role in the regulation of genome stability, energy homeostasis, stress signaling, and aging process [62,63]. Since sirtuins are a major consumer of NAD^+^, their strict correlation with vascular ageing and function is not surprising. An upregulation of SIRT1 in endothelium has been demonstrated to prevent the endothelial dysfunction in vivo and to counteract the increase in stiffness of the large arteries, thus preventing two key age-related changes in the vasculature [64]. Notably, individuals who are characterized by low SIRT1 levels early in life are prone to develop premature microvascular dysfunction during adulthood and display a higher risk of developing cardiovascular and cerebrovascular diseases [65]. Consistent with these findings, endothelial SIRT1-deficient mice are characterized by a marked endothelial dysfunction that promotes the development of micro- and macro-vascular complications in vivo [66].

On the contrary, another in vivo study demonstrated that the overexpression of SIRT1 in endothelial cells displayed protective activity against the age-induced impairment of vasodilator responses and inhibited vasoconstrictor responses to acetylcholine, suggesting that boosting the endothelial expression of SIRT1 may represent a promising intervention for treatment of cardiovascular diseases [67]. It has been reported that inhibition of SIRT1 in the endothelium inhibits the endothelium-dependent vasodilation and decreases the bioavailability of nitric oxide (NO) in small and large arteries both in in vitro and in vivo models, an effect associated with the deacetylation and subsequent activation of eNOS, which sustains vascular homeostasis through NO production [68]. Nonetheless, it is not clarified if the deacetylation of eNOS has any impact on its phosphorylation status and the following NO release, although the eNOS deficiency does not disrupt the vasoprotective effects of endothelial SIRT1 [69].

While the upregulation of SIRT1, consequent to caloric restriction, is reduced in eNOS knockout mice, the overexpression of human SIRT1 displays a protective effect against age-related adverse arterial remodeling of eNOS-deficient mice, thus indicating a complex reciprocal regulation in the endothelium [70].

Patients affected by cardiovascular and cerebrovascular diseases display mitochondrial dysfunction of endothelial cells, which is responsible for a depressed endothelium-dependent vasodilatation through decreased NO bioavailability, associated with high levels of hydrogen peroxide [71].

It has been reported that SIRT1 participates in the regulation of the biogenesis of mitochondria by increasing the expression of PPAR-ɣ coactivator-1α (PGC-1α) together with other genes, and the upregulation of SIRT1 is able to partially restore endothelium-dependent vasodilatation in mice characterized by impaired mitochondrial function [72]. Furthermore, it has been reported that during aging the decrease in SIRT1 expression observed in endothelium is associated with an upregulation of COX-2, which generates vasoconstrictors to boost endothelium-dependent contractions [67].

**Figure 2:**
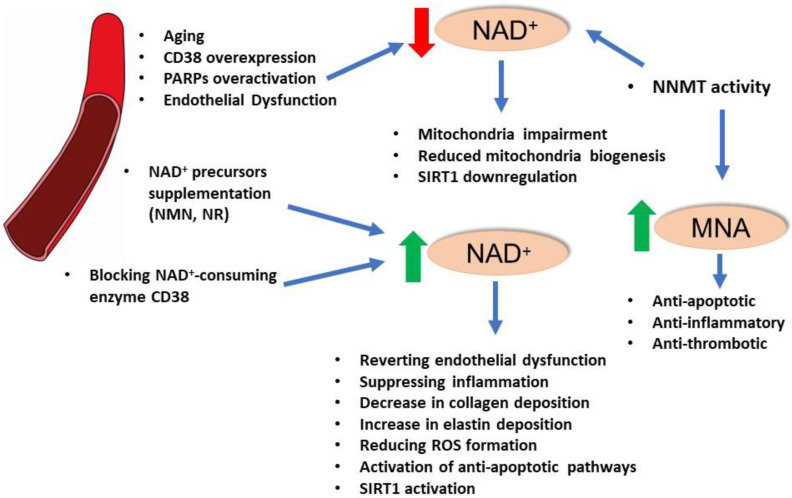
Impact of NAD^+^ homeostasis and major NAD^+^-consuming enzymes on vascular health.

It has been described that oxidative stress is one of the causes that induces a downregulation of SIRT1 expression in endothelium and a main source of endothelial dysfunction, while defects in endothelial SIRT1 activity can trigger an increase in ROS production and vascular inflammation [67,73,74,75]. Caloric restriction effectively counteracts SIRT1 arterial decline and endothelial dysfunction by decreasing the oxidative stress in in vivo animal models [76]. Since SIRT1 is a NAD^+^ sensor, it displays strong antioxidant capacities and pharmacological agents able to regulate SIRT1 signaling have been studied, and clearly SIRT1 function is highly influenced by the variations in intracellular NAD^+^ levels [77]. It has been hypothesized that during aging, the NAD^+^ level declines, partially due to the intensified activity of NAD-consuming enzymes, such as poly(ADP-ribose)-polymerases (PARPs). As mentioned in the previous paragraph, an approach aiming to boost SIRT1 function is to enhance the intracellular NAD^+^ levels by administering its precursors NMN and NR. Indeed, it has been reported that the use of NMN and NR exerts potent vascular antiaging effects in vivo, rescues endothelium-mediated vasodilation, and improves cerebral blood supply, and they are able to modulate endothelial NAD^+^ level [78,79]. Intriguingly, the strategy to enhance the NAD^+^ levels by stimulating its biosynthesis seems to be more effective in improving the vascular function in old mice rather than in young ones. However, it is important to notice that the responses were only partial and were not able to fully restore the endothelial and vascular function at a level comparable to young animals [80]. Nonetheless, there is a high interest in SIRT1-based vascular antiaging drugs for the treatment of cardiovascular and cerebrovascular diseases (Figure 2).

## 6. NAD^+^-Dependent Enzymes and Vascular Function: PARPs

PARPs have been widely studied due to their crucial role in DNA repair, inflammatory response, and cell death, although recently several reports have demonstrated that they might also play a role in circadian rhythm regulation, functionality of neurons, endoplasmic reticulum stress, metabolism, and many other crucial cellular pathways [81,82,83,84,85]. Poly(ADPribose) polymerase-1 (PARP1) is the most expressed among the several PARP isoforms and is responsible for more than 90% of the PARPs catalytic activity observed in the nucleus of cells [86]. Following finding a DNA-strand break, PARP1 binds to the DNA strand cleaving NAD^+^ between NA and ribose, yielding NAM, and subsequently modifies the DNA nuclear acceptor proteins triggering the creation of a bond between the protein and the ADP-ribose residue. This process yields ribosyl–ribosyl links, which are a signal for other DNA-repairing enzymes and DNA base repair response [87,88]. However, in the case of excessive DNA damage, an overactivation of PARPs will result in the depletion of NAD^+^ reserves, which in turn will impair the glycolysis rate, mitochondrial electron transport chain, and ultimately ATP production [89,90]. It has been demonstrated that extensive DNA breaking can dramatically reduce intracellular NAD^+^ content up to 20–30% of its normal level, resulting in severe limitation of NAD^+^ availability for other NAD^+^-dependent enzymes since PARP1 displays a very high affinity for NAD^+^ due to the Michaelis–Menten constant (K_m_) of 5.0 × 10^−5^ mol/L (Table 1) [91,92,93,94]. Since the K_m_ is a measure of how efficiently an enzyme converts a substrate into product, the low K_m_ value of PARP1 for NAD^+^ implicates that this enzyme is one of the major NAD^+^ consumers that competes with SIRT1 for the intracellular substrate.

Indeed, PARP1 knockdown in an in vivo model was shown to trigger a 2-fold increase in NAD^+^ content [95]. In the case of DNA damage, endothelial cells can follow three main pathways depending on the extent of the DNA damage and the degree of PARP activation. Upon a brief ischemic event, mild to moderate DNA injury can occur in endothelial cells, resulting in the activation of PARP1 together with other DNA repair machineries that fix the DNA damage and promote the survival of endothelial cells. On the contrary, an excessive DNA damage activates the second pathway of apoptotic endothelial cell death which caspases cleave PARPs, thus limiting or suppressing the ability of PARPs to respond to DNA breakages and allowing the preservation of intracellular ATP required for the endothelial cell apoptosis [96,97]. The third pathway is prompted by massive DNA injury and results in overactivation of PARPs, which depletes the intracellular reserve of NAD^+^, thus suppressing the activity of SIRT1. All these events impair the glycolysis, the Krebs cycle, and the mitochondrial electron transport chain, dramatically impacting the production of ATP and thus triggering endothelial cell death by necrosis [97].

**Table 1:** K_m_ values of the main NAD^+^-consuming enzymes.

Enzyme	K_m_ Value	Reference
CD38	1.5–2.5 × 10^−5^ mol/L	[98]
SIRT1	9.4–9.6 × 10^−5^ mol/L	[99]
PARP1	5.0 × 10^−5^ mol/L	[94]

Several studies have reported that extensive PARP1 activation contributes to maintaining vascular inflammation and endothelial dysfunction. Von Lukowicz et al. investigated the role of PARPs in atherogenesis through pharmacological PARP inhibition or genetic PARP1 deletion in atherosclerosis-prone apolipoprotein E-deficient mice (in vivo). This study demonstrated that inhibition of PARPs, as well as genetic deletion, triggered a significant decrease in plaque formation. Furthermore, it was demonstrated that PARP1 activation increases the expression of adhesion molecules and activates endothelial cells, promotes the infiltration of inflammatory cells, and induces features of plaque vulnerability, thus suggesting that inhibition of PARP1 may represent a promising therapeutic target in atherosclerosis [100]. Another study demonstrated an association between cell death and oxidative stress-associated DNA damage and PARP activation within atherosclerotic plaques in apolipoprotein E [ApoE] (^−^/^−^) model of high-fat diet-induced atherosclerosis. Indeed, PARP inhibition significantly reduced the number and size of plaques and altered the structural constitution of plaques without affecting sera lipid contents [101].

PARP inhibition increased the amount of collagen content via the stimulation of tissue inhibitor of metalloproteinase-2, stimulated the transmigration of smooth muscle cells to intima layer of the atherosclerotic plaque, and suppressed the production of monocyte chemotactic protein-1, all of which are widely recognized as markers of plaque stability [101]. These observations indicate that PARP inhibition may promote plaque stability. Furthermore, PARP inhibition may prevent plaque development by decreasing inflammatory factors and interfering with plaque dynamics-related cellular changes, thus being a promising strategy for the treatment of atherosclerosis [101]. Interestingly, PARP1^−^/^−^ mice (in vivo) are characterized by the downregulation of several transcription factors, stress-related, and diminished expression of genes encoding for cytokines and cellular adhesion molecules [102].

It has been demonstrated that PARP1 plays an important role in the pathogenesis of endothelial dysfunction in diabetes. The destruction of islet cells in mice upon administering streptozotocin led to hyperglycemia, intravascular oxidant production, DNA strand breakage, PARP activation, and a selective loss of endothelium-dependent vasodilation. In mice, the destruction of islet cells with streptozotocin triggered hyperglycemia, intravascular oxidative stress, DNA breaks, PARP activation, and a selective loss of endothelium-dependent vasodilation. In this context, high glucose-treated endothelial cells displayed increased the generation of reactive oxygen and nitrogen species as a consequence of single-strand DNA breaks, and PARP1 overactivation with associated metabolic and functional damage. High glucose triggered a significant PARP-dependent ATP decrease in endothelial cells and NADPH. Since eNOS is an NADPH-dependent enzyme, it was hypothesized that the observed depletion of NADPH in endothelial cells, which occurs upon high glucose exposure, directly inhibits eNOS activity and is the main cause of the selective suppression of endothelium-dependent vasodilation in the diabetic blood vessels [103]. In line with these findings, another study measured the levels of PARP1 activation in subjects equally distributed among healthy, parental history of type 2 diabetes, impaired glucose tolerance, and diabetic groups. Interestingly, the results obtained by the authors of this study demonstrated that PARP activation can be observed not only in established type 2 diabetic patients, but also in healthy subjects at risk of developing diabetes, and that PARP activation is associated with impairments in the vascular reactivity of the microcirculation of the skin [104]. Thus, taken together, these studies demonstrate that PARP1 is a promising target for the treatment of diabetic endothelial dysfunction.

## 7. Enzymes of NAD^+^ Homeostasis: Nicotinamide N-methyltransferase (NNMT)

NNMT is a cytosolic enzyme that plays a pivotal role in modulating cellular energy homeostasis by regulating NAM and S-adenosyl-L-methionine (SAM) flux within the critical NAD^+^ salvage pathway and methionine cycle, respectively [105]. In this context, the methylation reaction of NAM to 1-methylnicotinamide (MNA) catalyzed by the enzyme is critical for the destiny of NAM, since, once methylated, it cannot enter the NAD^+^ salvage pathway [106]. Indeed, it has been demonstrated that the inhibition of NNMT activity results in an increase of intracellular NAD^+^ levels, thus suggesting that a variation of NNMT activity can potentially have a great impact on NAD^+^-dependent enzymes [107]. NNMT is largely studied in cancer, where it was found to be upregulated in several types of solid tumors [108,109,110,111,112,113]. Recent studies demonstrated that NNMT protects endothelium from oxidant stress-induced injury, and the product of its reaction, the MNA, has been found to exert important vasoprotective effects [114,115,116,117]. Indeed, it has been demonstrated that MNA is able to reverse endothelial dysfunction via NO production, thus exerting a protective effect on aorta [118]. However, a subsequent study from the same group, although confirmed that MNA improved the survival of diabetic mice, reported that this effect was not associated with a correction of the parameters of long term glycemic control [119]. Nonetheless, MNA has been demonstrated to increase Tyr/Phe ratio in a in vivo model of hypercholesterolemic mice (ApoE/LDLR^−^/^−^) and improved endothelial function, and authors stated that the decreased levels observed for this ratio could reflect eNOS uncoupling, a source of oxidative stress which promotes endothelial dysfunction [120]. Furthermore, MNA administration has been proved to upregulate PGI2, which prevent platelets aggregation, proliferation of smooth muscle cells and leukocytes adhesion to the endothelium [121,122]. MNA displays anti-inflammatory functions consequent to the improvement of endothelial NO and PGI2 levels, since it has been demonstrated that its administration does not affect the reduction of inflammatory cytokines in macrophages [123]. Finally, MNA has been shown to also exert anti-thrombotic effect through release of PGI2 in studies, which was compared the anti-atherosclerotic effects of MNA and NA in a in vivo model of ApoE/LDLR^−^/^−^ mice. Moreover, MNA and NA administration was able to reduce plaque size, macrophage infiltration, and lipid content, including cholesterol, cholesteryl ester, and triglyceride [124]. Notably, the administration of NA has been shown to exert anti-inflammatory and cardio-protective effects upon being converted to MNA [124,125,126,127]. However, in ApoE/LDLR^−^/^−^ mice, the MNA administration displayed more potent reductive effects than NA on atherosclerotic plaques, a phenomenon that could be related to the SAM depletion which, in turn, can affect the epigenetics of the endothelial cells [106,124,128]. Altogether, these studies demonstrate that MNA exerts positive effects on endothelial function. However, the increased methylation activity of NNMT, which converts NA to MNA, can prevent NA to enter the NAD^+^ salvage pathway, thus resulting in decreased intracellular NAD^+^ levels, potentially boosting the negative effects associated to NAD^+^ decline [107]. Therefore, the use of NNMT inhibitors for the treatment of several pathological conditions should be carefully evaluated when considering the potential side effects on endothelium [129,130,131,132,133,134,135,136].

## 8. Conclusions

In recent years, several studies have focused on the impact of NAD^+^ homeostasis and NAD^+^-dependent enzymes dysregulation on vascular function. Moreover, many studies have demonstrated that counteracting the NAD^+^ decline observed with ageing can exert beneficial effects for the vasculature.

However, several difficulties and problems related to the administration of NAD^+^ precursors as therapeutics must be resolved in order to translate the experimental findings into clinical practice. An important issue is the optimal administration modality, since high doses of NAD^+^ precursors could cause hepatotoxicity and other harmful effects. At the current moment, both NMN and NR seem to be promising candidates for enhancing NAD^+^ levels in vascular cells, while administering NAM could be detrimental due to the negative feedback exerted by NAM on SIRT1, which displays critical positive effects on vasculature. For the same reason, the use of NNMT inhibitors, widely studied for anti-cancer applications, should be carefully evaluated in order to prevent negative effects consequent to SIRT1 inhibition. Furthermore, excessive NAD^+^ levels could, on the contrary, being harmful due to a consequent overactivation of PARPs.

In conclusion, targeting vascular NAD^+^ metabolism displays important therapeutic potential for the clinical management of age-related cardiovascular dysfunctions. Indeed, cardiovascular diseases are the major cause of morbidity, especially in the elderly, and are responsible, directly or indirectly, for about 30% of deaths globally. Thus, improving vascular health would have a great impact on morbidity of the population and may reduce the costs related to the health care of patients, especially the elderly.

Although future studies are required to better clarify the potential of targeting NAD^+^ homeostasis, the strategies based on administering NAD^+^ precursors or inhibiting NAD^+^-dependent enzymes seem to be an attractive approach for reducing chronic low-grade inflammation, enhancing mitochondrial biogenesis, and improving oxidative metabolism in vascular cells.

## Abbreviations

Nicotinamide adenine dinucleotide (NAD^+^); tryptophan (Trp); nicotinic acid (NA); nicotinamide (NAM); nicotinamide riboside (NR); nicotinic acid phosphoribosyltransferase (NAPRTase); 5-phosphoribosyl-1-pyrophosphate (PRPP); nicotinic acid mononucleotide (NAMN); pyrophosphate (PPi); nicotinamide mononucleotide adenylyltransferases1-3 (NMNATs1-3); nicotinic acid adenine dinucleotide (NAAD); NAD synthase (NADSYN); indoleamine 2,3-dioxygenase (IDO); tryptophan 2,3-dioxygenase (TDO); N-formylkynurenine (NFK); α-amino-β-carboxymuconate-ε-semialdehyde (ACMS); quinolinic acid (QUIN); quinolinate phosphoribosyltransferase (QPRT); nicotinamide phosphoribosyltransferase (NAMPT); nicotinamide riboside kinase (NRK); glyceraldehyde-3-phosphate (G3P); glyceraldehyde-3-phosphate dehydrogenase (GAPDH); tricarboxylic acid (TCA); cyclooxygenase (COX); endothelial nitric oxide synthase (eNOS); reactive oxygen species (ROS); nitric oxide (NO); PPAR-ɣ coactivator-1α (PGC-1α); poly(ADP-ribose)-polymerases (PARPs); Poly(ADPribose) polymerase-1 (PARP1); Nicotinamide N-methyltransferase (NNMT); S-adenosyl-L-methionine (SAM); 1-methylnicotinamide (MNA).
